# Evaluation of Oxidative Profiling in Semen Fractions and Its Relationship With Sperm Function in Kangal Akkaraman Rams

**DOI:** 10.1111/rda.70295

**Published:** 2026-08-13

**Authors:** Abdulkadir Kaya, Omer Varisli, Ali Senol, Haceli Ocal, Hamide Taskin, Bora Ozarslan

**Affiliations:** ^1^ Department of Reproduction and Artificial Insemination, Faculty of Veterinary Medicine Kırıkkale University Kırıkkale Turkey; ^2^ Department of Biochemistry, Faculty of Veterinary Medicine Kırıkkale University Kırıkkale Turkey; ^3^ Ministry of Agriculture and Forestry Ankara Turkey; ^4^ Department of Animal Breeding and Husbandry, Faculty of Veterinary Medicine Kırıkkale University Kırıkkale Turkey

**Keywords:** malondialdehyde, oxidative stress, protein normalization, ram semen, seminal plasma, sperm pellet fraction, viability

## Abstract

The determination of sperm oxidative stress provides significant information related to semen quality. Most studies investigate oxidative stress markers only at the level of seminal plasma, but this approach may provide an incomplete overview of the oxidative status of semen. The aim of the study is to determine the relationship between protein‐normalized oxidative stress markers and spermatological parameters in ram semen fractions. A total of 38 ejaculates obtained from 24 rams were fractionated into seminal plasma, whole semen (non‐centrifuged), and pellet at different concentrations. Total antioxidant status (TAS), total oxidant status (TOS), malondialdehyde (MDA), and oxidative stress index (OSI) were measured and normalized to total protein content using the Lowry method. Fraction‐wise differences in oxidative stress parameters were analysed using linear mixed‐effects models accounting for ram identity, while relationships between spermatological and oxidative parameters were evaluated using Spearman correlation analysis. Principal component analysis (PCA) and hierarchical clustering were performed as exploratory multivariate visualization tools. Motility and viability were positively correlated (*ρ* = 0.856, *p* < 0.001), while both parameters were negatively correlated with abnormal sperm rate (*p* < 0.05). In addition, TOS and MDA levels were significantly lower in seminal plasma than in sperm pellet fractions, supporting the presence of fraction‐dependent oxidative profiles (*p* < 0.05). Exploratory PCA suggested that overall multivariate variation was mainly associated with spermatological parameters, whereas oxidative markers had a more limited contribution. These findings provide insight into the relationship between oxidative status and different semen fractions. Additionally, fraction‐specific oxidative profiling may provide complementary information beyond seminal plasma measurements alone.

## 1 Introduction

During the process from ejaculation to fertilization, sperm needs high energy levels for motility, ion exchange in the cell membrane, capacitation, hyperactivation, and the acrosome reaction. This energy demand is achieved largely through mitochondrial oxidative phosphorylation, and this reaction leads to the production of reactive oxygen species (ROS) (Raad et al. 2024; Xu et al. 2025). At the physiological level, ROS can be tolerated by spermatozoa. They are even required for capacitation through the modulation of membrane fluidity, ion fluxes, and protein phosphorylation pathways. However, high ROS production leads to oxidative stress, resulting in lipid peroxidation, protein oxidation, DNA fragmentation, and decreased motility (Aitken and Drevet 2020; O'Flaherty and Scarlata 2022).

Ram spermatozoa are sensitive to oxidative damage due to their high content of polyunsaturated fatty acids within the plasma membrane (Güngör et al. 2024; Hamilton et al. 2016). Furthermore, cryopreservation may alter membrane lipid composition, cholesterol content, and antioxidant status, thereby increasing sensitivity to lipid peroxidation and membrane disruption (Güngör et al. 2024). Lipid peroxidation has been shown to reduce motility and increase DNA fragmentation in ram semen (Hamilton et al. 2016). Also, alterations in membrane integrity are directly associated with fertility ability in rams (Bodu et al. 2025). Oxidative balance means the interaction between oxidant production and antioxidant defence (Sies et al. 2022). Sperm are protected against oxidative stress by antioxidants. These antioxidants are enzymatic, such as Superoxide Dismutase (SOD), Catalase (CAT), and Glutathione Peroxidase (GPx), and non‐enzymatic, such as vitamin E, vitamin C, and glutathione (Kowalczyk 2022; Wang et al. 2025). In ram semen, seminal plasma is an important source of antioxidants, whereas spermatozoa exhibit low antioxidant capacity (Martí et al. 2007; Catalán et al. 2022). This situation becomes critical during dilution and cryopreservation because the reduced level of seminal plasma increases oxidative stress (Riesco et al. 2021).

Oxidative status is frequently evaluated using Total Antioxidant Status (TAS), Total Oxidant Status (TOS), and Malondialdehyde (MDA). TAS indicates total antioxidant capacity and TOS shows the total oxidant load. MDA is a lipid peroxidation product that indicates membrane damage. The Oxidative Stress Index (OSI), calculated as TOS/TAS, provides a measure of oxidative stress (Denk et al. 2026; Ünal and Uysal 2024). However, most studies in ram semen evaluate single biomarkers or single fractions, often without protein normalization or multivariate evaluation (Subramanian et al. 2018; Vakili et al. 2025). Methods such as principal component analysis and clustering are limited in their ability to demonstrate oxidative balance (Rhouma et al. 2025). Furthermore, most studies have used only seminal plasma to evaluate oxidative stress, whereas semen pellet fractions are often ignored (Catalán et al. 2022; Martí et al. 2007; Subramanian et al. 2018; Ünal and Uysal 2024; Vakili et al. 2025).

In sheep farms, sperm quality and oxidative stress status are key determinants of male fertility and reproductive efficiency. Reductions in spermatological parameters are associated with infertility and lower pregnancy rates (Aitken and Drevet 2020; Amann and Hammerstedt 2002). In addition, oxidative damage may further compromise reproductive outcomes and embryo development (Sakkas and Alvarez 2010). These impairments can reduce lambing rates and offspring yield, thereby decreasing flock productivity, while prolongation of the lambing season may also create significant management challenges.

The present study aimed to evaluate spermatological parameters (volume, concentration, motility, viability, and abnormal sperm ratio) and protein‐normalized oxidative stress markers (TAS, TOS, MDA, and OSI) in semen samples obtained from 24 rams. In addition to seminal plasma and non‐centrifuged semen, sperm pellet fractions were adjusted to increasing sperm concentrations to evaluate whether the protein‐normalized oxidative profile of the cellular sperm component differs among fractions. Oxidative parameters were normalized to total protein content using the Lowry method to allow comparisons among semen fractions. Principal component analysis and hierarchical clustering were used only as exploratory tools to visualize multivariate relationships among oxidative parameters and semen quality variables.

## 2 Materials and Methods

### 2.1 Animals

A total of 38 semen samples were collected from 24 clinically healthy Kangal Akkaraman rams aged 2–4 years with a body condition score of 3.0–3.5 (5‐point scale) at the Ulaş Agricultural Enterprise Directorate (TİGEM), Sivas, Türkiye (39°26′26″ N, 37°01′46″ E). Rams were maintained under standardized housing and feeding conditions. Ethical approval for this study was obtained from Kırıkkale University.

### 2.2 Semen Collection and Initial Evaluation

Semen samples were collected using an artificial vagina. A maximum of one ejaculate per day was obtained from each ram; if a second collection was required, a minimum interval of 2 days was maintained. Immediately after collection, semen samples were maintained at 37°C in a water bath until evaluation (Aibazov et al. 2022).

#### 2.2.1 Sperm Volume and Concentration

Ejaculate volume was recorded immediately after collection by measuring the semen directly in a graduated glass collection tube with a precision of 0.1 mL (Aibazov et al. 2022). Sperm concentration was determined using a hemocytometric method (Thoma counting chamber) under a light microscope (Kaya et al. 2026).

#### 2.2.2 Motility

Sperm motility was assessed using a phase‐contrast microscope (Leica DM1000, Germany) equipped with a heated stage maintained at 37°C. Semen samples were diluted with phosphate‐buffered saline (PBS) and examined at 400× magnification. Motility percentages were recorded in at least three separate microscopic fields, following the method described by Varışlı et al. (2018).

#### 2.2.3 Sperm Viability and Abnormal Morphology

Sperm viability and abnormal morphology were evaluated using eosin–nigrosin staining technique. Briefly, 5 μL of semen was mixed with the stain, smeared onto clean glass slides, air‐dried, and examined under a light microscope. A total of 200 spermatozoa per smear were assessed. Spermatozoa exhibiting unstained heads were considered viable, whereas those with pink‐stained heads were classified as non‐viable. Morphological abnormalities were recorded as head defects, midpiece defects, tail defects, and the presence of cytoplasmic droplets (Bhalothia et al. 2022). Reference ranges for all spermatological parameters described are presented in Table 1.

**TABLE 1:** Reference ranges for fresh ram semen quality parameters.

Parameter	Range	References
Volume (mL/ejaculate)	0.6–1.0	Acısu et al. (2025), Hossain et al. (2018)
Concentration (×10^9^ sperm/mL)	1.5–3.0	Petrean et al. (2023), Acısu et al. (2025), Riesco et al. (2020)
Motility (%)	80%–90%	Vašíček et al. (2022), Petrean et al. (2023), Acısu et al. (2025)
Viability (%)	78%–85%	Vašíček et al. (2022), Bodu et al. (2025), Acısu et al. (2025)
Abnormal sperm ratio (%)	3%–5%	Bodu et al. (2025), Petrean et al. (2023), Acısu et al. (2025)

### 2.3 Semen Fractionation Protocol

Semen was processed immediately at 4°C and separated into distinct fractions. One aliquot from each ejaculate was kept as non‐centrifuged semen (S). The remaining semen sample was centrifuged at 3000 × g for 10 min to separate seminal plasma and the sperm pellet. The supernatant was collected as seminal plasma (SP). The resulting sperm pellet was resuspended in PBS and adjusted to final sperm concentrations of 50, 100, 200, 300, and 400 × 10^6^ spermatozoa/mL, representing sperm pellet fractions P5, P10, P20, P30, and P40, respectively. Thus, the experimental fractions consisted of non‐centrifuged semen (S), seminal plasma (SP), and five sperm pellet fractions with increasing sperm concentrations (P5–P40). All prepared fractions were aliquoted and stored at −80°C until biochemical analyses.

The sperm pellet fractions were generated to evaluate the protein‐normalized oxidative profile of the cellular sperm component at increasing sperm concentrations and to determine whether oxidative stress markers differed among seminal plasma, non‐centrifuged semen, and sperm‐rich fractions. Since all fractions were derived from the same ejaculate, the fractionation protocol allowed comparison of oxidative stress markers across different semen components while preserving the biological relationship among fractions.

On the day of analysis, frozen aliquots were thawed, and 100 μL aliquots from each fraction, including non‐centrifuged semen, seminal plasma, and sperm pellet suspensions, were mixed with 900 μL PBS. Seminal plasma samples were diluted in the same manner but were not subjected to ultrasonic homogenization because they did not contain a cellular sperm pellet. Non‐centrifuged semen and sperm pellet suspensions were transferred into a 2 mL beaker placed in ice water and homogenized using a probe‐type ultrasonic homogenizer (Bandelin Sonopuls, Geräte‐Typ: UW 2070, Berlin, Germany). Sonication was performed in amplitude‐control mode at 25% amplitude for 10 s and repeated six times with 30‐s intervals between cycles. During the procedure, samples were kept in ice water to minimize heat‐induced oxidative changes. The prepared homogenates and diluted seminal plasma samples were then used for total protein determination and oxidative stress analyses (Varışlı and Akyol 2019).

### 2.4 Determination of Oxidative Stress Markers

Total protein concentrations were determined using the Lowry method. Briefly, homogenized samples were mixed with alkaline copper reagent, followed by the Folin–Ciocalteu reagent, and incubated at room temperature. Absorbance was measured at 750 nm using a Thermo Scientific Multiskan GO microplate spectrophotometer. Bovine Serum Albumin (BSA) was used to generate a standard curve (*R* ^2^ > 0.99), and all samples were analysed in duplicate (Lowry et al. 1951).

#### 2.4.1 Total Antioxidant Status (TAS)

Total antioxidant capacity was measured using a commercial ABTS‐based colorimetric kit (Elabscience, E‐BC‐K801‐M). In this method, antioxidants in the sample reduce the pre‐formed ABTS•^+^ radical, resulting in a decrease in colour intensity proportional to total antioxidant activity. Optical density was measured at 660 nm with a microplate reader, and TAS values were calculated using a Trolox calibration curve. Data were expressed as mmol Trolox equivalent per mg protein.

#### 2.4.2 Total Oxidant Status (TOS)

Total oxidant status (TOS) was determined using a commercial colorimetric assay kit (Elabscience, E‐BC‐K802‐M). The method is based on the oxidation of ferrous ion–o‐dianisidine complex to ferric ion by oxidants present in the sample, forming a coloured complex measurable spectrophotometrically. Absorbance was measured using a microplate reader, and results were expressed as μmol H₂O₂ equivalents per mg protein.

#### 2.4.3 Malondialdehyde (MDA)

Lipid peroxidation was assessed by measuring MDA levels using the thiobarbituric acid (TBA) reaction. Samples were reacted with TBA reagent and incubated in a boiling water bath for 1 h. After cooling and centrifugation, the absorbance of the supernatant was measured at 535 nm using a spectrophotometer. MDA concentration was calculated using the molar extinction coefficient (1.56 × 10^5^ mol^−1^ cm^−1^) and expressed as nmol per mg protein (Banday et al. 2017).

#### 2.4.4 Oxidative Stress Index (OSI)

The oxidative stress index (OSI) was calculated as the ratio of TOS to TAS, as previously described by Ünal and Uysal (2024). OSI reflects the balance between total oxidant load and antioxidant capacity in semen samples.

$$\begin{array}{c} \mathrm{OSI}\;\left(\text{arbitrary unit}\right)=\mathrm{TOS}\;\left(\text{μmol}\;H₂O₂\text{equivalent}/L\right)\\ /\mathrm{TAS}\;\left(\text{mmol Trolox equivalent}/L\right)\times 100 \end{array}$$

### 2.5 Statistical Analysis

Descriptive statistics, normality assessment, and correlation analyses were performed using GraphPad Prism 10 (GraphPad Software Inc., San Diego, CA, USA). Data normality was assessed using the Shapiro–Wilk test. Descriptive statistics for the overall dataset are presented as mean ± standard deviation (SD) together with minimum–maximum values to summarize the general characteristics of the study population and to allow comparison with conventional semen quality reports. In contrast, fraction‐wise oxidative stress parameters are presented as median with interquartile range (IQR) because these variables were not normally distributed. Relationships among spermatological and oxidative stress variables were evaluated using Spearman rank correlation analysis.

For fraction‐wise comparisons of oxidative stress parameters, linear mixed‐effects models were performed using IBM SPSS Statistics 15.0 (IBM Corp., Armonk, NY, USA) to account for the animal effect arising from repeated ejaculates obtained from some rams. Semen fraction was included as a fixed effect, whereas ram identity was included as a random effect. Separate models were constructed for TOS, TAS, MDA, and OSI. Post hoc pairwise comparisons among semen fractions were performed using Bonferroni adjustment. Although fraction‐wise data are presented as median (IQR), statistical significance and superscript letters in Table 4 were assigned according to the mixed‐effects model results. Statistical significance was set at *p* < 0.05.

Principal component analysis (PCA) and hierarchical clustering heatmap analysis were conducted using the MetaboAnalyst 6.0 platform (www.metaboanalyst.ca) as exploratory multivariate analyses. Prior to multivariate analysis, variables were autoscaled by mean‐centering and division by standard deviation to account for differences in measurement units and variable ranges. PCA was used to visualize the overall multivariate distribution of ejaculates in a score plot, with sperm motility retained as a continuous variable and displayed as a colour gradient. PCA loadings were examined to identify the variables contributing most strongly to the principal components. Hierarchical clustering heatmap analysis was performed using Euclidean distance and Ward's linkage method, with sperm motility included as a continuous annotation.

## 3 Results

Descriptive statistics are presented in Table 2. Mean ejaculate volume was 1.20 ± 0.25 mL, while sperm motility, viability, abnormal sperm rate, and concentration averaged 56.00% ± 16.59%, 62.43% ± 19.87%, 6.13% ± 3.51%, and 2.55 ± 0.78 × 10^9^/mL, respectively. Oxidative stress parameters showed mean values of 20.78 ± 7.54 μmol H₂O₂ eq./mg protein for TOS, 0.490 ± 0.223 mmol Trolox eq./mg protein for TAS, 0.308 ± 0.083 nmol/mg protein for MDA, and 1.174 ± 0.364 for OSI. Overall, oxidative stress parameters exhibited relatively great variability compared to spermatological parameters, indicating heterogeneity in oxidative status among samples.

**TABLE 2:** Overall descriptive statistics of spermatological parameters measured in whole semen samples and protein‐normalized oxidative stress parameters measured across all semen fractions, including non‐centrifuged semen (S), seminal plasma (SP), and sperm pellet fractions (P5–P40).

Parameter	Mean ± SD	Min–Max
Semen Volume (mL)	1.20 ± 0.25	0.50–1.70
Sperm Motility (%)	56.00 ± 16.59	16.70–83.30
Sperm Viability (%)	62.43 ± 19.87	23.50–98.10
Abnormal sperm ratio (%)	6.13 ± 3.51	0.00–15.00
Sperm Concentration (×10^9^/mL)	2.55 ± 0.78	1.00–4.10
TOS (μmol H₂O₂ eq./mg protein)	20.78 ± 7.54	2.64–33.62
TAS (mmol Trolox eq./mg protein)	0.490 ± 0.223	0.005–0.842
MDA (nmol/mg protein)	0.308 ± 0.083	0.073–0.490
OSI (arbitrary unit)	1.174 ± 0.364	0.051–2.958

*Note:* Values are presented as mean ± standard deviation (SD) with minimum and maximum values to summarize the overall dataset. Min–Max indicates minimum and maximum values.

Abbreviations: MDA, malondialdehyde; OSI, oxidative stress index; TAS, total antioxidant status; TOS, total oxidant status.

Correlation analysis demonstrated a strong positive association between motility and viability (*ρ* = 0.856, *p* < 0.001; Table 3). Both motility and viability were negatively correlated with abnormal sperm rate (*p* < 0.05). Semen volume showed moderate positive correlations with motility and viability (*p* < 0.05). Among oxidative stress markers measured in non‐centrifuged semen, TOS was positively correlated with MDA and OSI, while TAS showed a weak positive correlation with MDA (*p* < 0.05).

**TABLE 3:** Spearman correlation analysis among oxidative stress and spermatological parameters.

Variable 1	Variable 2	*ρ* (Spearman)	*p*
MOT	VIA	0.856	< 0.001
MOT	ABN	−0.504	0.001
VIA	ABN	−0.391	0.015
VOL	MOT	0.368	0.023
VOL	VIA	0.367	0.023
S‐TOS	S‐MDA	0.504	0.001
S‐TOS	S‐OSI	0.474	0.003
S‐TAS	S‐MDA	0.460	0.004

*Note:* Only statistically significant correlations (*p* < 0.05) are presented. *ρ*: Spearman correlation coefficient.

Abbreviations: ABN, abnormal sperm rate; MOT, motility; S‐MDA, malondialdehyde level measured in non‐centrifuged semen; S‐OSI, oxidative stress index calculated for non‐centrifuged semen; S‐TAS, total antioxidant status measured in non‐centrifuged semen; S‐TOS, total oxidant status measured in non‐centrifuged semen; VIA, viability; VOL, semen volume.

Linear mixed‐effects model analysis showed significant fraction effects for all oxidative stress parameters (*p* < 0.001; Table 4). TOS values were lowest in SP, intermediate in S, and higher in all pellet fractions. TAS values were also lowest in SP and lower in S than in pellet fractions, although some differences were observed among pellet fractions. MDA values were lowest in SP and higher in pellet fractions, with P30 showing the highest level. In contrast, OSI was higher in SP than in S and pellet fractions, while S and pellet fractions did not differ significantly from each other. These results indicate that oxidative stress parameters differed among semen fractions after accounting for ram identity.

**TABLE 4:** Comparison of protein‐normalized oxidative stress parameters among non‐centrifuged semen, seminal plasma, and sperm pellet fractions.

Group	TOS	TAS	MDA	OSI
SP	5.26 (2.63–7.61)^c^	0.018 (0.005–0.037)^d^	0.136 (0.073–0.269)^e^	1.207 (0.051–3.922)^a^
S	17.00 (11.77–25.43)ᵇ	0.410 (0.229–0.616)^c^	0.276 (0.176–0.395)^d^	0.986 (0.528–1.653)^b^
P5	24.09 (21.36–28.90)^a^	0.633 (0.439–0.827)^a^	0.315 (0.273–0.368)^cd^	1.200 (0.900–1.493)^b^
P10	25.65 (16.41–31.21)^a^	0.569 (0.429–0.755)^ab^	0.343 (0.269–0.398)^bc^	1.251 (0.785–1.825)^b^
P20	23.92 (18.07–33.26)^a^	0.619 (0.403–0.842)^a^	0.340 (0.307–0.393)^bc^	1.161 (0.685–1.898)^b^
P30	25.07 (18.18–32.96)^a^	0.563 (0.401–0.748)^b^	0.387 (0.316–0.490)^a^	1.291 (0.774–1.823)^b^
P40	23.73 (15.73–33.62)^a^	0.539 (0.345–0.730)^b^	0.341 (0.282–0.434)^b^	1.144 (0.807–1.548)^b^

*Note:* Values are presented as median (IQR). Different superscript letters within a column indicate significant differences (*p* < 0.05). Overall fraction effects were significant for all oxidative stress parameters based on linear mixed‐effects models with ram identity included as a random effect (*p* < 0.001). Post hoc pairwise comparisons were performed using Bonferroni adjustment.

Abbreviations: MDA, malondialdehyde; OSI, oxidative stress index; P5–P40, sperm pellet concentration groups; S, non‐centrifuged semen; SP, seminal plasma; TAS, total antioxidant status; TOS, total oxidant status.

**FIGURE 1:**
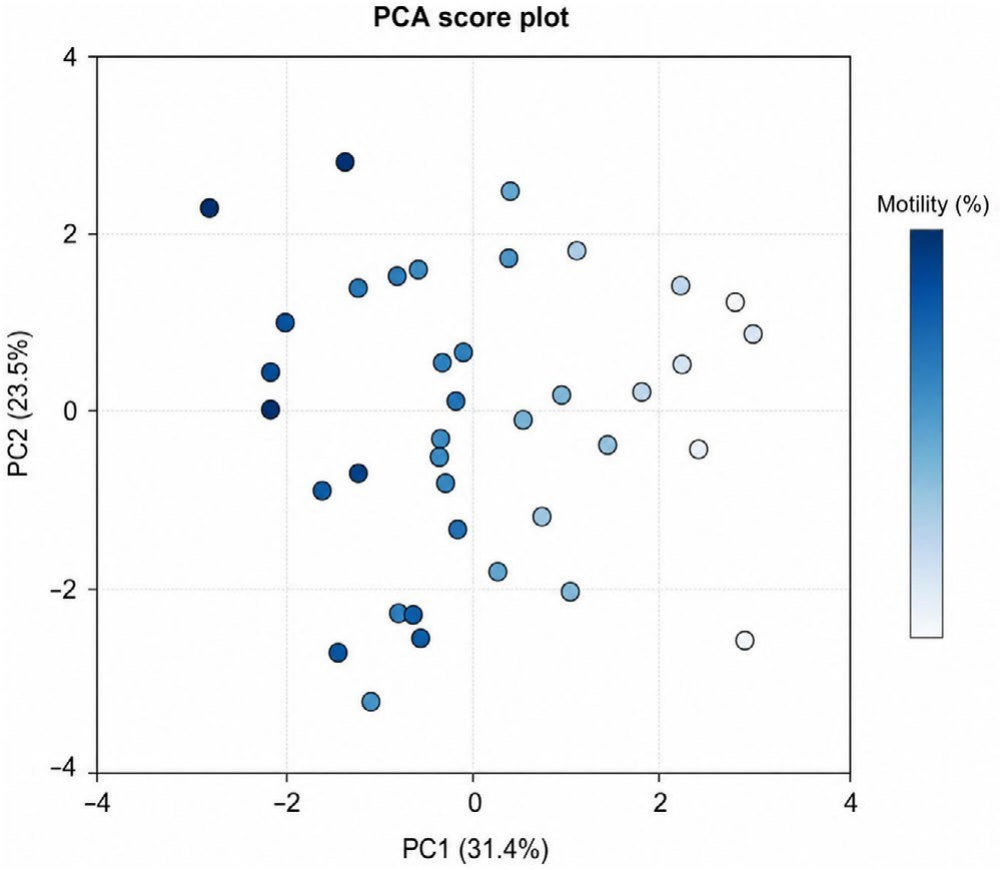
Principal component analysis (PCA) score plot of ejaculates based on autoscaled spermatological and whole‐semen oxidative stress variables. Each point represents one ejaculate, and the colour gradient indicates sperm motility as a continuous variable. PC1 and PC2 explained 31.4% and 23.5% of the total variance, respectively.

**FIGURE 2:**
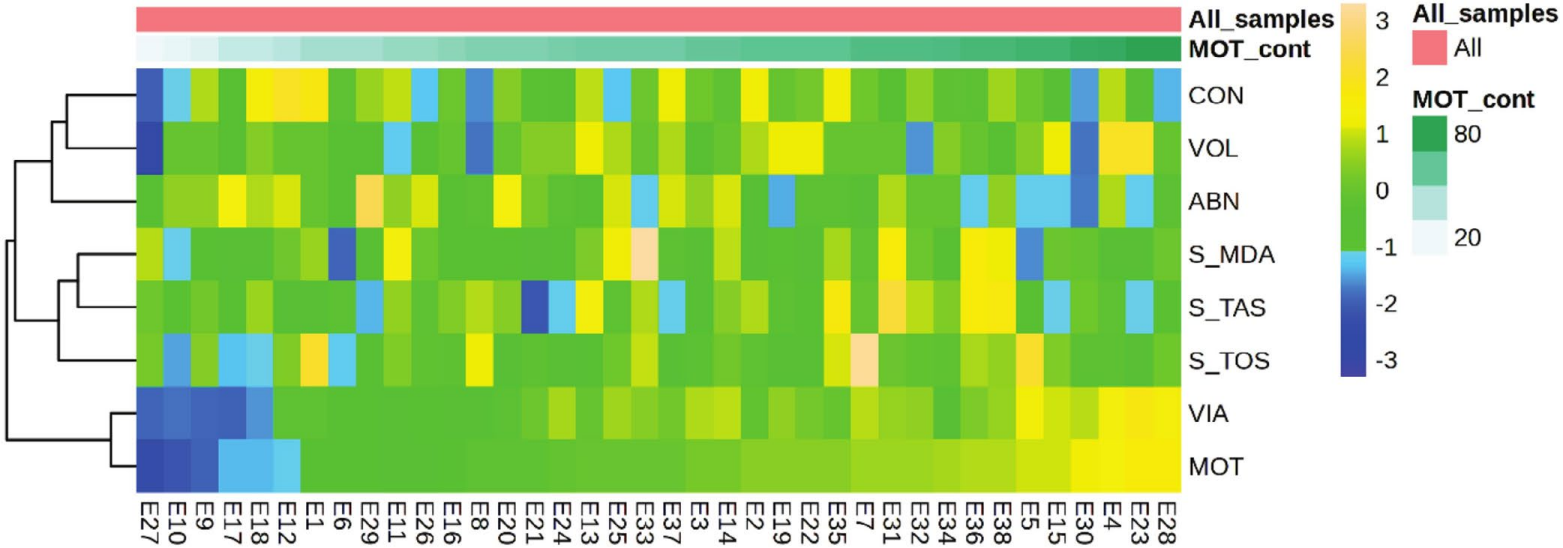
Hierarchical clustering heatmap of ejaculates based on autoscaled spermatological and whole‐semen oxidative stress variables. The variables included volume (VOL), sperm concentration (CON), motility (MOT), viability (VIA), abnormal sperm ratio (ABN), whole‐semen total oxidant status (S_TOS), whole‐semen total antioxidant status (S_TAS), and whole‐semen malondialdehyde level (S_MDA). Variables were clustered using Euclidean distance and Ward's linkage method. Sperm motility was included as a continuous annotation (MOT_cont).

Exploratory multivariate analyses were performed to visualize the relationships among spermatological parameters and oxidative stress markers measured in S. In the PCA score plot, PC1 and PC2 explained 31.4% and 23.5% of the total variance, respectively (Figure 1). PCA and heatmap analyses suggested that spermatological parameters contributed more prominently to the overall multivariate pattern than oxidative stress markers (Figures 1 and 2).

## 4 Discussion

The present study demonstrates that oxidative balance in ram semen varies among semen fractions and is related to sperm function. Motility and viability showed a strong positive correlation. Fraction‐wise analysis demonstrated that oxidative stress markers differed among semen components after accounting for ram identity. Oxidant levels and lipid peroxidation were higher in sperm pellet fractions than in seminal plasma, whereas OSI remained stable across fractions. These findings highlight the importance of protein‐normalized oxidative measurements for understanding the relationship between oxidative status and ram sperm quality.

When compared with previously reported reference ranges for ram semen quality (Acısu et al. 2025; Bodu et al. 2025; Petrean et al. 2023; Riesco et al. 2020; Vašíček et al. 2022; Table 1), sperm motility and viability values in the present study were lower, while the abnormal sperm rate was slightly higher. These differences may be primarily attributed to the heterogeneity of the sample population. In this study, semen samples were collected from many animals (24 rams), which likely increased biological variability. In contrast, many previous studies have been conducted using a smaller number of animals with repeated collections, resulting in more homogeneous datasets (Petrean et al. 2023; Vašíček et al. 2022; Bodu et al. 2025). Therefore, the variability may reflect a more realistic representation of population‐level semen quality rather than repeated conditions. This variability may influence reproductive performance, fertilization, pregnancy rates, and flock productivity.

The study demonstrated a positive relationship between motility and viability and a negative relationship with abnormal sperm rate. These findings indicate a central mechanism underlying sperm quality, and a similar pattern has also been demonstrated in other studies (Leugoué et al. 2022; Vakili et al. 2025). However, other studies have shown a positive correlation between motility and viability with enzymatic antioxidants such as TAS, SOD, and GPx, while it is negatively correlated with MDA and abnormal sperm rate (Tabatabaei Vakili et al. 2025; Mehdipour et al. 2025). These findings indicate that oxidative damage has a direct effect on spermatological parameters. Findings based on flow‐cytometric analyses performed on ram semen indicate that although motility and viability are frequently related, motile sperm are not always viable, and these two parameters reflect different aspects of sperm physiology (Vašíček et al. 2022). While our data revealed a strong correlation between motility and viability, this correlation should not be interpreted as universally valid under all biological conditions and in different samples. Also the positive association between TOS and MDA reflects the expected link between oxidative burden and lipid peroxidation (Ayala et al. 2014; Wu et al. 2017; Zińczuk et al. 2019), the concurrent increase in TAS alongside MDA suggests a compensatory antioxidant response to elevated oxidative stress (Table 3). This finding shows that redox balance is dynamic, and antioxidant capacity may increase together with oxidative damage rather than following a simple inverse relationship.

Although oxidative stress markers were not the dominant contributors in the exploratory multivariate analyses, they remain biologically relevant to semen quality. Excessive ROS production can impair sperm membrane integrity, mitochondrial function, and lipid stability, leading to lipid peroxidation and reduced sperm function (Barik et al. 2019; Sengupta et al. 2024). In this context, MDA is commonly considered an indicator of oxidative membrane damage, while antioxidant capacity may reflect a compensatory protective response. Previous studies in ram semen have also shown that antioxidant supplementation can improve spermatological parameters (Barik et al. 2019; Leugoué et al. 2022; Mehdipour et al. 2025; Tabatabaei Vakili et al. 2025). However, the relationships between oxidative markers and sperm function are likely complex and not strictly linear. This may explain why oxidative stress markers showed weaker associations with sperm quality parameters in the present study.

Unlike most previous studies that have assessed oxidative stress primarily in seminal plasma as a marker of redox status (Martí et al. 2007; Subramanian et al. 2018; Catalán et al. 2022; Ünal and Uysal 2024; Vakili et al. 2025), the present study evaluated oxidative markers not only in seminal plasma but also in non‐centrifuged semen and sperm pellet fractions at different concentrations. Seminal plasma is widely recognized as an important antioxidant reservoir of semen, containing enzymatic and non‐enzymatic defence systems that buffer ROS (Martí et al. 2007; Catalán et al. 2022). However, spermatozoa have limited cytoplasmic antioxidant capacity and are highly susceptible to lipid peroxidation due to their polyunsaturated fatty acid‐rich membranes (Aitken and Drevet 2020; Wang et al. 2025). Methodological work has previously suggested that appropriate homogenization and pellet‐specific analysis may help detect cellular oxidative markers more accurately (Varışlı and Akyol 2019). In the present study, TOS and MDA levels were lower in seminal plasma and higher in sperm pellet fractions, indicating that the cellular sperm component has a distinct oxidative profile. However, this finding should not be interpreted as evidence that seminal plasma analysis is insufficient in all cases. Rather, fraction‐specific and protein‐normalized oxidative profiling may provide complementary information beyond seminal plasma measurements alone. This approach may be particularly useful in future in vitro studies, including antioxidant supplementation, sperm toxicology, and other experimental models in which spermatozoa may be directly affected.

OSI is considered an integrative indicator of redox balance rather than absolute oxidant load alone because it reflects the relationship between oxidant production and antioxidant capacity (Ünal and Uysal 2024; Barik et al. 2019). Previous studies have shown that elevated seminal plasma OSI may be negatively associated with sperm motility, viability, and cryotolerance (Barranco et al. 2021; Catalán et al. 2022). In the present study, OSI showed a different pattern from individual oxidative markers. Although TOS and MDA levels were higher in sperm pellet fractions, OSI was higher in seminal plasma, while non‐centrifuged semen and pellet fractions showed similar OSI values. This finding may be explained by the proportional relationship between oxidant load and antioxidant capacity, since OSI is calculated from TOS and TAS. Therefore, OSI should be interpreted together with individual oxidative markers rather than as a standalone indicator of oxidative damage. This interpretation is also consistent with the concept that redox balance is dynamic and depends on both ROS production and antioxidant defence capacity (Sies et al. 2022).

Significant associations between oxidative stress markers and spermatological parameters have been reported in different species and clinical settings. For example, MDA levels have been negatively associated with sperm concentration and motility, whereas antioxidant enzymes such as CAT, SOD, and GPx have been positively related to sperm quality; increased oxidative stress has also been linked to abnormal sperm morphology (Rhouma et al. 2025). In male infertility, oxidative damage involves coordinated alterations in ROS production, lipid peroxidation, and antioxidant defence systems, indicating that single‐marker assessment may provide an incomplete view of redox status (Ayad et al. 2022). Similarly, integrative biomarker panels have been suggested to provide stronger diagnostic information than isolated biochemical markers in clinical studies (Bergsma et al. 2022). In the present study, correlation, PCA, and hierarchical clustering analyses were used to describe the relationship between oxidative status and semen quality variables in ram semen. However, these analyses should be interpreted as exploratory rather than confirmatory. Overall, our findings suggest that sperm quality in rams is associated with a coordinated redox profile rather than isolated oxidative markers alone.

This study has several limitations. The sample size was moderate and limited to clinically healthy rams from a single enterprise, which may limit how well the findings apply to other breeds, management systems, or production conditions. In addition, the cross‐sectional design of the study allows evaluation of associations between oxidative markers and sperm function, but does not permit causal interpretation. Oxidative assessment was limited to TAS, TOS, MDA, and OSI. Including antioxidant enzymes, mitochondrial oxidative stress indicators, or DNA damage markers could provide more detailed insight. Another limitation is that semen samples were collected under field‐laboratory conditions at the Ulaş Agricultural Enterprise, where access to advanced semen analysis equipment was limited. Therefore, sperm motility was assessed subjectively by phase‐contrast microscopy rather than by computer‐assisted sperm analysis (CASA). Although the available analyses were performed under standardized conditions, CASA would provide a more objective and detailed evaluation of sperm motility, including progressive motility and kinematic parameters. Therefore, motility‐related interpretations should be considered with caution. In addition, PCA and hierarchical clustering analyses were used only as exploratory visualization tools to describe general multivariate patterns among selected variables; therefore, these results should not be interpreted as confirmatory evidence of functional impairment or biologically distinct semen quality groups. Finally, fertility outcomes were not measured, which limits how well the results reflect in vivo reproductive performance.

## 5 Conclusions

This study, based on 38 ejaculates from 24 rams, showed that oxidative balance varies among semen fractions and that markers measured in cellular fractions are associated with sperm function. Oxidative values were lower in seminal plasma but higher in sperm‐rich pellet fractions, suggesting that seminal plasma alone may underestimate intracellular oxidative stress. Although TOS, TAS, and MDA differed among fractions, OSI remained relatively stable, indicating a balance between oxidants and antioxidants. Protein normalization using the Lowry method enabled reliable comparisons across fractions. Future research should integrate objective analysis using CASA and flow‐cytometric assessments, and expanded redox panels including specific antioxidant enzymes, mitochondrial ROS, and nitrosative stress markers (e.g., nitric oxide derivatives and peroxynitrite). Such multidimensional approaches would further clarify the mechanistic links between cellular redox dynamics and functional sperm competence in rams.

## Author Contributions

Conceptualization: A.K., O.V. Methodology: A.K., O.V. Investigation: A.K., O.V., A.S., H.O., B.O., H.T. Data curation: A.K., A.S. Formal analysis: A.K. Visualization: A.K. Writing – original draft: A.K. Writing – review and editing: A.K., O.V., A.S. Supervision: O.V. All authors have read and approved the final version of the manuscript.

## Conflicts of Interest

The authors declare no conflicts of interest.

## Data Availability

The data that support the findings of this study are available from the corresponding author upon reasonable request.
